# The bowel preparation for magnetic resonance enterography in patients with Crohn’s disease: study protocol for a randomized controlled trial

**DOI:** 10.1186/s13063-018-3101-x

**Published:** 2019-01-03

**Authors:** Min Dai, Ting Zhang, Qianqian Li, Bota Cui, Liyuan Xiang, Xiao Ding, Rong Rong, Jianling Bai, Jianguo Zhu, Faming Zhang

**Affiliations:** 1 Medical Center for Digestive Diseases, the Second Affiliated Hospital of Nanjing Medical University, 121 Jiang Jia Yuan, Nanjing, 210011 China; 2 Key Lab of Holistic Integrative Enterology, Nanjing Medical University, 121 Jiang Jia Yuan, Nanjing, 210011 China; 3 Department of Gastroenterology, the Second Affiliated Hospital of Xuzhou Medical University, Xuzhou, 221000 China; 4 Department of Biostatistics, School of Public Health, Nanjing Medical University, Nanjing, 211100 China; 5 Department of Radiology, the Second Affiliated Hospital of Nanjing Medical University, Nanjing, 210011 China; 6 Division of Gastroenterology, Sir Run Run Shaw Hospital, Nanjing Medical University, Nanjing, 211166 China

**Keywords:** Magnetic resonance enterography, Crohn’s disease, Bowel preparation, Transendoscopic enteral tubing, Colonoscopy

## Abstract

**Background:**

Adequate bowel preparation is required for magnetic resonance enterography (MRE), which can be achieved by administering contrast solution after mid-gut tubing or taking contrast solution orally. We present the design of randomized controlled trial (RCT) to compare the efficacy and compliance of bowel preparation between mid-gut tubing and oral administering for MRE in patients with Crohn’s disease (CD).

**Methods/design:**

This is an open-label, multicenter RCT. Ninety-six patients with CD in need of MRE examination and mid-gut tubing (prepared for fecal microbiota transplantation and/or enteral nutrition), aged ≥ 14 years, will be included. Patients will be randomized 1:1 into either bowel preparation by oral administering (oral group) or bowel preparation through mid-gut transendoscopic enteral tubing (TET) (tubing group). The primary outcome measures are: (1) degree of discomfort before/during/after bowel preparation for MRE using a visual 5-grade scale (1 = few, 5 = very severe); and (2) grade of bowel distention evaluated by a 5-grade scale (1 = 0–20% segmental distention, 2 = 20–40% distention, 3 = 40–60% distention, 4 = 60–80% distention, 5 = 80–100% distention). The secondary outcome measure is the accuracy of lesion detection through MRE confirmed by colonoscopy which is evaluated by a 5-point scale.

**Discussion:**

The outcome of this study is expected to provide a novel effective clinical protocol of bowel preparation for MRE in patients with CD. We hope to highlight the concept of physician–patient satisfaction based on different methods of bowel preparation for MRE.

**Trial registration:**

ClinicalTrials.gov, NCT03541733. Registered on 30 May 2018.

**Electronic supplementary material:**

The online version of this article (10.1186/s13063-018-3101-x) contains supplementary material, which is available to authorized users.

## Background

Crohn’s disease (CD) is a chronic inflammatory disorder that may invade the gastrointestinal tract from mouth to anus; it is characterized by periods of flare-up with active symptomatic disease and periods of remission [1]. Inflammation of CD is transmural and therefore may be complicated by fistula, abscess formation, perforations, and fibrotic strictures. The frequent and periodic evaluations of inflammation of CD are vital in planning a proper therapy, monitoring the drug effects, and detecting recurrence [2].

Magnetic resonance enterography (MRE), a target examination of the gastrointestinal tract, has been shown to be highly effective in the diagnosis and management of patients with CD [3]. A recent systematic review indicated that the sensitivity and specificity of MRE for the diagnosis of suspected CD were 78% and 85%, respectively [4]. For the extension of CD lesions, the sensitivity and specificity of MRE for small bowel lesions were 74% and 91%, respectively [4]. On a per-patient basis, MRE had an overall sensitivity of 91% and a specificity of 71% for active disease [5]. Compared with other imaging examinations, MRE has many advantages such as no ionizing radiation, offering better soft tissue contrast resolution, superior evaluation of perianal disease, better distinction between acute and chronic disease, distinguishing from prominent muscle hypertrophy to prominent fibrosis [6], and superior detection of fistulas and strictures in CD with functional techniques such as diffusion-weighted magnetic resonance imaging (DW-MRI) and dynamic contrast-enhanced MRI (DCE-MRI) [7].

MRE examination requires adequate bowel distention as collapsed loops may hide lesions or mimic disease by suggesting a thickened bowel wall [8]. Bowel distention can be achieved by two methods, administering contrast solution after mid-gut tubing and administering contrast solution orally. Mid-gut tubing, such as nasojejunal tubing and nasoduodenal tubing, provides better bowel distention [9, 10]. Traditionally, mid-gut tubing can be operated under fluoroscopic or electromagnetic guidance or with endoscopic assistance. The procedure of conventional tubing is considered to be unpleasant and time-consuming, and/or with radiation in patients especially younger ones [10]. The transendoscopic enteral tubing (TET) in mid-gut is a novel and quick technique of enteral tubing under the endoscopy [11]. The mean procedure time (from the beginning of inserting the tube into the esophagus to the tube being fixed on the pylorus wall by one titanium clip) for tubing was 4.2 ± 1.9 min (range, 1.53–11.25 min) [11]. The mid-gut tube can be used for repeat fecal microbiota transplantations (FMTs) and enteral nutrition support in CD [12–14]. Furthermore, based on our practice, we found that the mid-gut tube might be used as a perfect delivering for the large volume laxative and contrast solution for bowel preparation for MRE. Bowel preparation for MRE includes bowel cleaning and bowel distention. Administering solution through the mid-gut tube may lead to better bowel distention and alleviates adverse symptoms from drinking a large volume of fluid laxative and contrast solution. In clinical practice, some patients may not tolerate a large oral fluid load, leading to adverse symptoms such as nausea, vomiting, bloating, abdominal pain, and diarrhea [15–17]. In addition, despite large oral volumes, distention of the distal small bowel, where diseases are most likely to occur, can still be poor [5]. This not only aggravates the mental pressure of patients, but also affects the accuracy of the judgment from the physicians for the disease. Therefore, our study aims to evaluate the efficacy and compliance of bowel preparation through mid-gut tubing for MRE in patients with CD.

## Methods/design

**Fig. 1:**
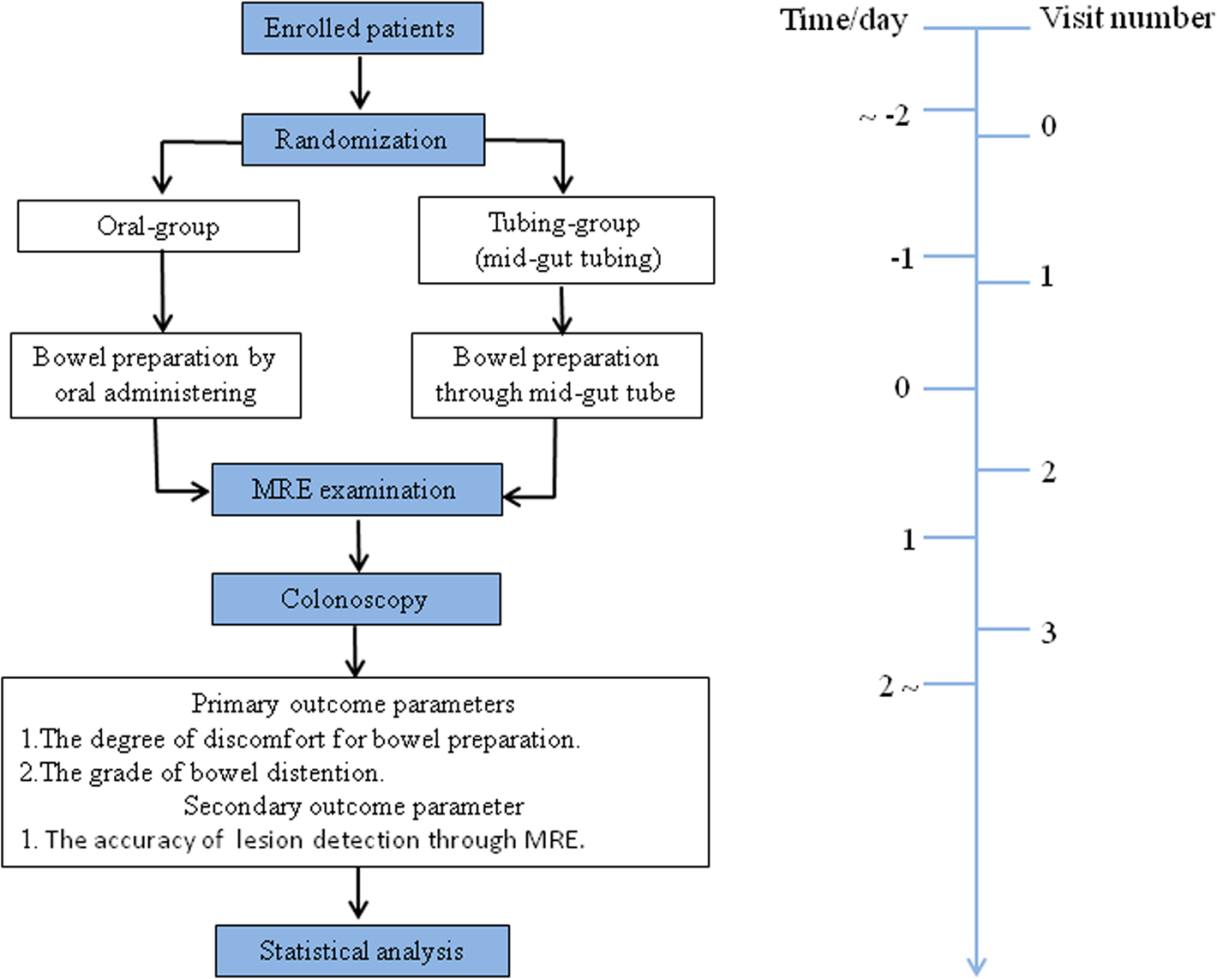
*Flow chart* of the trial

**Fig. 2:**
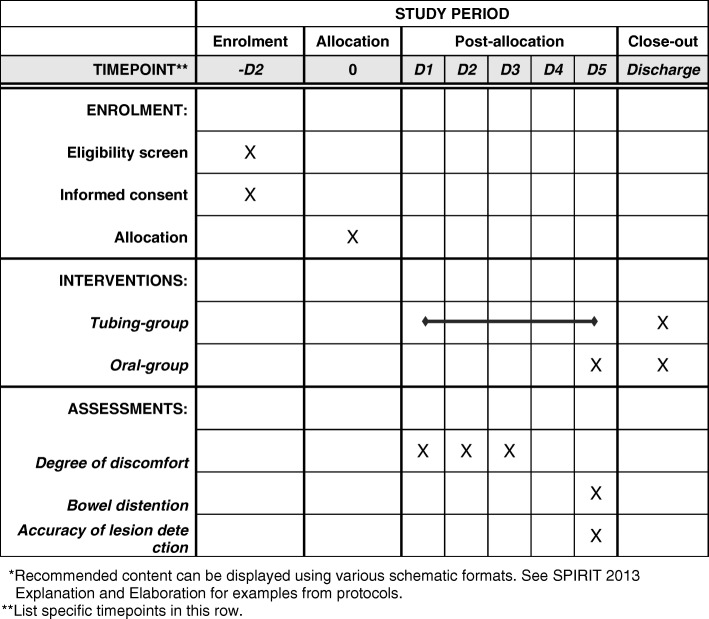
SPIRIT figure

This study is conducted in China as an open-label, multicenter RCT with a parallel group design. Flow chart of the trial is shown in Fig. 1. The procedure and checklist of this trial are shown in SPIRIT figure (Fig. 2) and Additional file 1: SPIRIT checklist, respectively.

### Participants

Participants will be recruited to the study from the gastroenterology inpatients at the Second Affiliated Hospital of Nanjing Medical University, Sir Run Run Shaw Hospital at Nanjing Medical University and the Second Affiliated Hospital of Xuzhou Medical University. The physicians will ask eligible patients at a routine visit whether they would consider participating in the study. If the patient agrees to participate, declaration of informed consent is signed. A screening visit is carried out to ensure that the patients meet the criteria for participation.

Inclusion criteria consist of: (1) patients with CD needing a MRE examination and mid-gut tubing (prepared for FMT and/or enteral nutrition); and (2) aged ≥ 14 years. Exclusion criteria consist of: (1) being unable to understand or provide informed consent; (2) having difficulty in swallowing or dysphagia; (3) being allergic to laxative and/or contrast; (4) being claustrophobic or pregnant or with implanted metal objects or a cardiac pacemaker precluding performance of MRI; and (5) having a known or suspected intestinal obstruction or severe stricture.

### Randomization

Randomized sequences will be generated by the statistician from Department of Biostatistics, Nanjing Medical university. SAS 9.4 software will be used to generate a random sequence table for the participants. Ninety-six random numbers and the allocation sequence table of the randomized group will be kept as blind codes. The participants will be randomly divided into the oral group (n = 48) and tubing group (n = 48) at a ratio of 1:1. This sequence of randomization will be contained in opaque sealed envelopes and kept by the clinical research coordinator, who will be contacted competitively by each physician to provide the allocation. The principal physicians from the above three centers are responsible for enrolling patients.

### Oral group

**Fig. 3:**
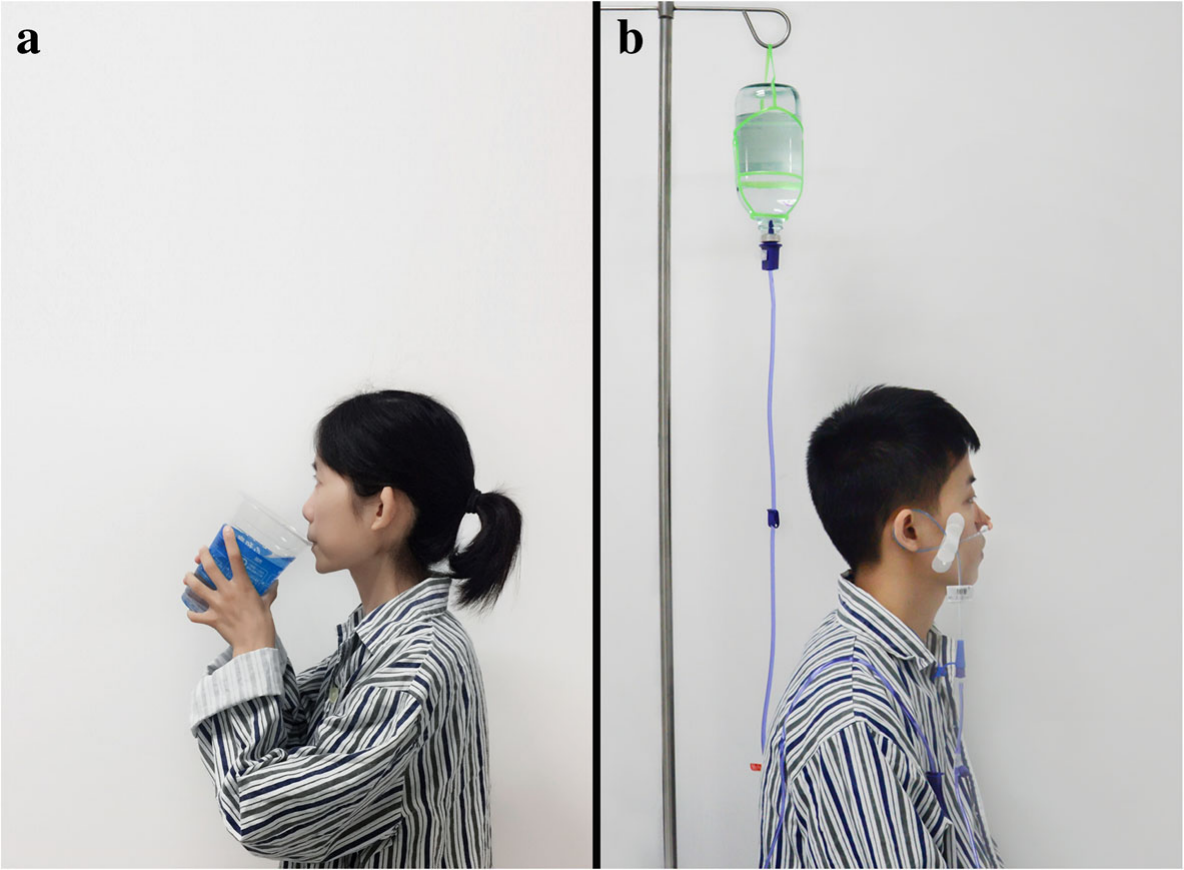
Methods of bowel preparation. Bowel preparation for MRE through oral administering (**a**) and mid-gut tubing (**b**)

Patients will be instructed to drink 2000 mL polyethylene glycol (PEG) solution over 60 min on the night before the MRE to remove stool and other impurities which may mimic lesions in the MRE. In addition, they will drink 1500 mL 3% mannitol solution gradually in the course of 60 min before MRE. An example of bowel preparation for MRE through oral administering is shown in Fig. 3a. If a patient fails to complete bowel preparation because of severe adverse symptoms such as nausea, vomiting, bloating, and abdominal pain and/or refusing to choose “oral” method, a tube will be placed in his or her mid-gut for MRE. Patients in the oral group will have a soft tube placed in the mid-gut after MRE for enteral nutrition or FMT.

### Tubing group

Patients will be inserted with a soft tube named mid-gut TET tube (8F, FMT-DT-N-27/1350, FMT Medical, Nanjing, China) in the mid-gut through endoscopy under anesthesia before the bowel preparation for MRE. If patients cannot tolerate endoscopy or anesthesia, a nasojejunal tube will be inserted under digital fluoroscopy. Patients will be instructed to take in 2000 mL PEG solution through the tube over 60 min on the night before MRE. In addition, 1500 mL 3% mannitol solution will be administered through the tube gradually in the course of 60 min before MRE to induce bowel distention. An example of bowel preparation for MRE through mid-gut tubing is shown in Fig. 3b. If a patient refuses the assigned mid-gut tubing before the MRE, or the tube falls off or blocks before the bowel preparation, the patient will be given the option of an alternative method.

All patients will have a colonoscopy examination within 24 h after the MRE. No change in the medical treatment will be made in this study until the colonoscopy examination is finished.

### MRE technique

All enrolled patients will be sent to the MRI department after bowel distention is induced. MRE examination will be performed using a 3.0-T clinical scanner (Signa HDxt, GE Healthcare) equipped with abdominal-pelvic coil (eight radiofrequency channels). Immediately before the scan, all patients will be given 20 mg of scopolamine-N-butyl bromide (Busco-pan; BoehringerIngelheim, Ingelheim, Germany) intravenously, to reduce bowel peristalsis motion artifacts. Patients will be scanned in the supine position. The MRE is carried out following the protocol [18, 19], including: (1) coronal T2 (single shot fast spin-echo [SSFSE]) through the abdomen and pelvis with breath-holding (Tck = 5 mm, spacing = 1 mm; TR = 2800 ms, TE = 70 ms); (2) axial T2 FSE fat-suppressed images covering the abdomen and pelvis, free-breathing with navigator triggering (Tck = 4 mm, spacing = 2 mm; TR = 12,000 ms, TE = 90 ms); and (3) axial T1 LAVA-Flex Mask through the abdomen and pelvis with breath-holding (Tck = 4 mm, spacing = 0 mm; TR = 4500 ms, TE = 1.7 ms). DW-MRI and DCE-MRI will be carried out after MRE. The whole imaging process takes approximately 45–50 min in total.

### Procedures

Over the one-week study period, the included patients will be instructed to answer a questionnaire about their mental pressure and discomfort (such as nausea, vomiting, bloating, abdominal pain, and diarrhea) before/during/after the bowel preparation for MRE. An overview of corresponding assessments is showed in Table 1. Two experienced radiologists will evaluate the grade of bowel distention. Two experienced endoscopists will evaluate the colonoscopy results.

**Table 1:** Summary of measures to be collected

Variable				
Visit number/bowel preparation	0/Screening	1/Before	2/During	3/After
Day	~ − 3	− 1	0	1~3
Female-sex (%)	A	–	–	–
Age (years) (mean ± SD)	A	–	–	–
Height (mean ± SD)	A	–	–	–
Weight (mean ± SD)	A	–	–	–
Disease duration (years) (mean ± SD)	A	–	–	–
Disease location (n)	A	–	–	–
Small bowel disease	A	–	–	–
Small bowel + colonic disease	A	–	–	–
Upper GI	A	–	–	–
Perianal disease	A	–	–	–
Previous CD-related surgery (n)	A	–	–	–
HBI	A	–	–	–
Current medication (n)	A	–	–	–
Systematic corticosteroids	A	–	–	–
Thiopurines	A	–	–	–
Mesalazine	A	–	–	–
Anti-TNF	A	–	–	–
Mental pressure (median)	–	A	–	–
Degree of discomfort (median)	–			
Nausea	–	A	A	–
Vomiting	–	A	A	–
Bloating	–	A	A	
Abdominal pain	–	A	A	A
Diarrhea	–	–	–	A
Distention grade (median)				
Jejunum	–	–	A	–
Ileum, proximal	–	–	A	–
Ileum, distal	–	–	A	–
Colon (right part)	–	–	A	–
Colon (left part)	–	–	A	–
Preference to the method (n)	–	–	–	A
MRE lesion detection	–			
Terminal ileum	–	–	–	A
Ileocecal junction	–	–	–	A
Hepatic flexure of colon	–	–	–	A
Splenic flexure of colon	–	–	–	A
Rectosigmoid colon	–	–	–	A

*A* assessed, − not assessed, *SD* standard deviation, *GI* gastrointestinal, *CD* Crohn’s disease, *HBI* Harvey Bradshaw Index, *TNF* tumor necrosis factor

Mental pressure and degree of discomfort: using a visual 5-grade to describe (1 = few, 5 = very severe); distention grade of bowel segments: using a 5-grade scale (1 = 0–20% segmental distention, 2 = 20–40% distention, 3 = 40–60% distention, 4 = 60–80% distention, 5 = 80–100% distention); ileum, distal (the last 20–25 cm of terminal ileum). MRE lesion detection is evaluated by a 5-point scale (lesions locating at the terminal ileum, ileocecal junction, hepatic flexure of colon, splenic flexure of colon, and rectosigmoid colon, consistency of lesion detection from each bowel segment scoring 1 point, otherwise not scoring, confirmed by colonoscopy)

### Outcome measures

Table 1 lists the outcome measures and the time points at which they are collected during the study.

### The primary outcome

**Fig. 4:**
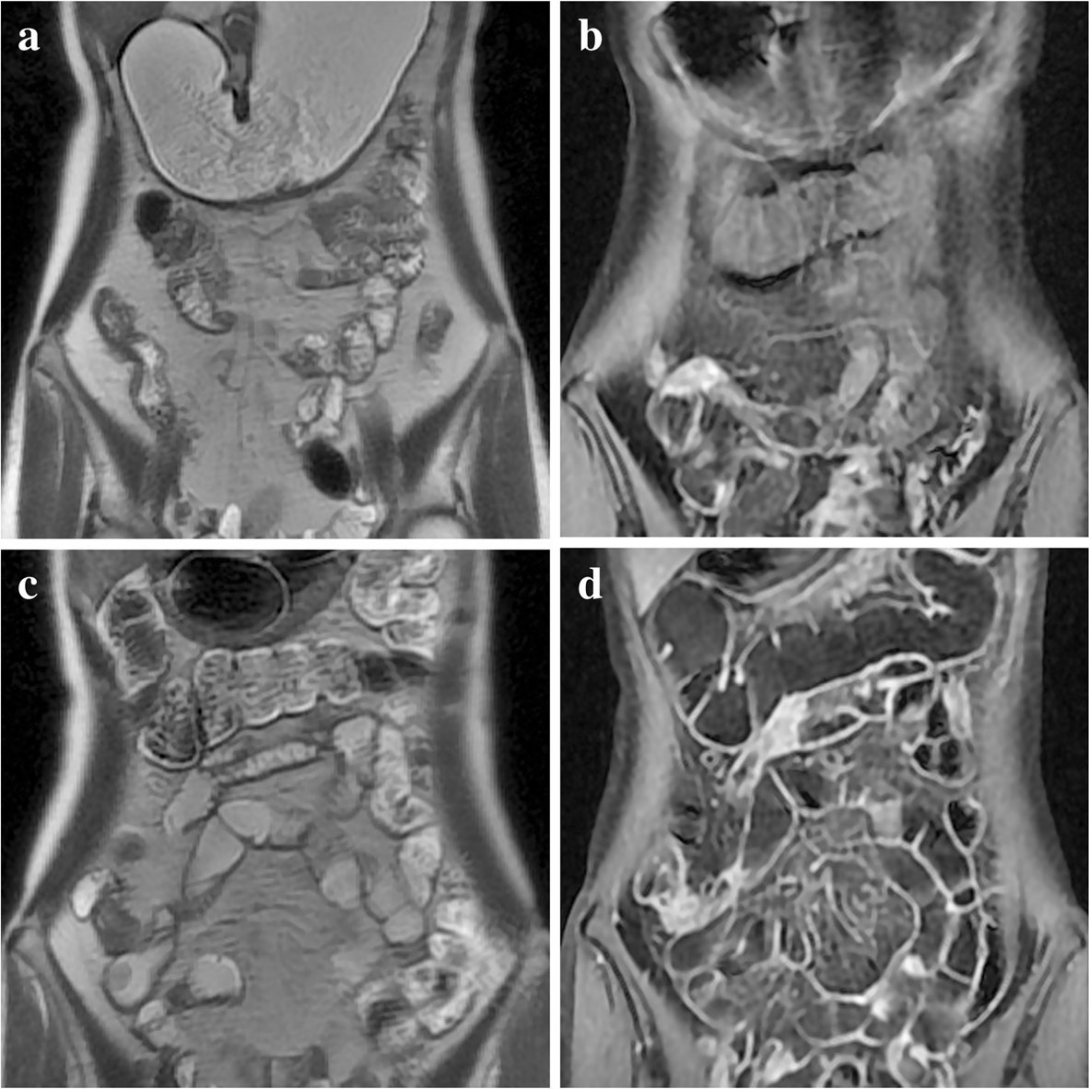
MR images. A 30-year-old woman with CD for eight years has a big polyp located at the duodenum inducing luminal stricture. Coronal T2-weighted SSFSE MRE (**a**, **c**) and coronal T1-weighted enhanced MR (**b**, **d**) are shown above. **a** and **b** are prepared through oral administering: retention of large volume contrast solution in the stomach and collapsed bowel segments representing terrible bowel distention. **c** and **d** are prepared through mid-gut tubing: good distention of small bowel

The primary outcome measures are: (1) degree of discomfort before/during/after bowel preparation for MRE using a visual 5-grade scale (1 = few, 5 = very severe). The bowel preparation includes two procedures, bowel cleaning and bowel distention, during which we use a visual 5-grade scale to describe the severity of nausea, vomiting, bloating, and abdominal pain (1 = few, 5 = very severe), respectively [20]. After the MRE, we use a visual 5-grade scale to describe the severity of abdominal pain and diarrhea. A lower score of adverse symptoms means a better compliance of the patient for bowel preparation for MRE; (2) grade of bowel distention evaluated by a 5-grade scale (1 = 0–20% segmental distention, 2 = 20–40% distention, 3 = 40–60% distention, 4 = 60–80% distention, 5 = 80–100% distention) [17, 21–23]. The distention grades of bowel segments, including jejunum, proximal ileum, distal ileum, right part colon, and left part colon, are assessed by two experienced radiologists, both of whom will be blinded to the information of allocation along above 5-grade scale. Different grades of bowel distention in the MR images achieved through two different methods are shown in the Fig. 4.

### The secondary outcome

The secondary outcome measure is the accuracy of lesion detection through MRE confirmed by colonoscopy over a 5-point scale (lesions locating at the terminal ileum, ileocecal junction, hepatic flexure of colon, splenic flexure of colon, and rectosigmoid colon, consistency of lesion detection from each bowel segment scoring 1 point, otherwise not scoring). A higher score of distention grade of bowel segments and accuracy of lesion detection represents a better efficacy of bowel preparation for MRE.

### Sample size calculation

The principal analysis will consist of the comparison between the proportions of bowel preparation that have reached physician–patient satisfaction score (degree of discomfort score ≤ 10, out of 50; grade of bowel distention score ≥ 20, out of 25; lesions detection point ≥ 4, out of 5) through mid-gut tubing and taking contrast compounds orally. Group sample sizes of 48 in the tubing group and 48 in the oral group achieve 80% power to detect a difference between the group proportions of 0.2500. The proportion in the tubing group is assumed to be 0.6000 under the null hypothesis and 0.8500 under the alternative hypothesis. The proportion in the oral group is 0.6000. The test statistic used is the two-sided Z test with unpooled variance. The significance level of the test was targeted at 0.0500. The significance level actually achieved by this design is 0.0506.

### Statistical analysis plan

Considering the evaluation for grade of bowel distention, we will calculate the mean, standard deviation, and range of distention scores for each bowel segments and total segments between the two groups by using the Wilcoxon rank sum test. Similarly, we will use the same methods to analyze adverse symptom severity and the accuracy of lesion detection between the two groups. We will compare the proportion of bowel preparation for MRE reaching physician–patient satisfaction score between two groups by using the Chi-square test as our primary analysis. We will also explore the correlations between grade of bowel distention and severity of adverse symptoms by using Spearman’s correlation coefficient. The patients’ preference between the two methods for MRE will be compared using the signed-rank test. All statistical analysis will be done using SPSS software (version 19.0; SPSS Inc., Chicago, IL). *P* < 0.05 will be considered statistically significant.

## Discussion

It has been claimed that inflammation of CD involves the entire gastrointestinal tract. Ileocolonoscopy is useful for detecting inflammation in colon and the terminal ileum. However, this technique cannot detect the inflammation in whole bowel. Recently, new endoscopic techniques have been developed, such as capsule endoscopy (CE) and balloon-assisted enteroscopy (BAE). These techniques have been used for the diagnosis and assessment of disease extent and severity in CD. Because the inflammation of CD is typically transmural, cross-section imaging techniques such as computed tomographic enterography (CTE) and MRE might be superior to endoscopy in visualization of extramural abnormalities such as fistulas and abscesses. The major disadvantage of CTE, however, compared with MRE, is its reliance on ionizing radiation. The mean CT dose index volume for multidetector CTE has been reported to be 4.9 mGy (range, 1.29–12.95 mGy) [24]. Although this dose has decreased in recent years with advances such as iterative reconstruction, it is still higher than that of ionizing radiation-free MRE. In addition, MRE has superior evaluation of perianal disease [25, 26], which is one of the most common and characteristic complications of CD.

Adequate bowel distention during MRE is crucial for the correct evaluation of bowel wall pathologies [27]. Studies have reported that there are two methods of bowel distention—contrast solution administration through nasojejunal tubing and ingestion of oral contrast solution—and a few studies made comparisons between the two methods. Negaard et al. [9] compared the two methods for MRE and demonstrated that bowel distention was superior in nasojejunal tubing. The diameter of the small bowel was larger in MRE after nasojejunal tubing than MRE through administering oral contrast compounds (difference jejunum: 0.55 cm, *P* < 0.001; ileum: 0.35 cm, *P* < 0.001, terminal ileum: 0.09 cm, *P* = 0.08). Masselli et al. [10] compared 22 patients underwent MRE through duodenal tubing with 18 patients through oral administration and demonstrated better bowel distention through duodenal tubing. MRE after duodenal tubing compared with MRE through administering oral contrast was statistically better when visualizing superficial abnormalities (*P* < 0.01). However, Schreyer et al. [28] did not find any significant difference in bowel distention in 21 patients with CD undergoing both two methods of MRE. The procedure of conventional mid-gut tubing for MRE is often the most traumatic part of the examination from the patients’ point of view, which is also considered time-consuming. In addition, one of the methods of tubing has not eliminated radiation exposure. Besides, the whole duration of the examination procedure cannot be separated with a MR compatible pump. The pump was used to enable patients ingest contrast compounds at an infusion rate of 80–150 mL/min. This infusion rate may be so fast that it leads to sever adverse symptoms such as nausea, vomiting, bloating, and abdominal pain [20].

In our study, we will use a novel and quick technique of enteral tubing under gastroscopy named mid-gut TET [11]. In our previous study, 86 patients underwent mid-gut TET and the success rate of the procedure was 98.8% (85/86) [11]. In addition, 97.7% (84/86) of patients were satisfied with the procedure of placement [11]. This mid-gut tube can serve as the delivery way of FMT, enteral nutrition support, and laxative agents and contrast solution for MRE bowel preparation. The laxative agents and contrast solution infusion rate is about 30 mL/min on average. A pump is not needed in the procedure of bowel preparation for MRE through mid-gut tube. Patients can prepare everything by themselves following the physicians’ instructions. They simply need to hang the bottle or sack for laxative agents and contrast solution on the infusion support and fully open the click for controlling the infusion rate. As observed from the pilot study, patients having difficulty in drinking large volumes of solution benefited from mid-gut tubing, with adverse symptoms alleviated or removed. This result is contrary to Negaad’s study probably because of the infusion rate of contrast solution. The infusion rate in Negaad’s study was 120–150 mL/min [20], which is almost 4–5 times of that in our study.

Above all, we assume that the novel, more acceptable, and more time-saving mid-gut tubing for MRE can lead to better bowel distention and less adverse symptoms, and subsequently higher accuracy of lesion detection to reach physician–patient satisfaction. This study aims to provide evidence to support the assumption. Physician–patient satisfaction emphasizes the fact that physicians and patients are satisfied with the accuracy of lesion detection and the reduction of pain, respectively. The results of this study are expected to provide an important basis for the clinical protocol of bowel preparation for MRE in patients with CD.

### Trial status

In total, we have currently included 29 patients in this trial at the time of submission of the revised protocol to *Trials* (20 November 2018). The first patient was included at the Second Affiliated Hospital of Nanjing Medical University.

## Additional file

Additional file 1: SPIRIT checklist. (DOC 117 kb)

## Abbreviations

BAE: Balloon-assisted enteroscopy
CD: Crohn’s disease
CE: Capsule endoscopy
CTE: Computed tomographic enterography
DCE-MRI: Dynamic contrast-enhanced magnetic resonance imaging
DW-MRI: Diffusion-weighted magnetic resonance imaging
FMT: Fecal microbiota transplantation
MRE: Magnetic resonance enterography
PEG: Polyethylene glycol
RCT: Randomized controlled trial
TET: Transendoscopic enteral tubing
