# Supplementary material for: The bowel preparation for magnetic resonance enterography in patients with Crohn’s disease: study protocol for a randomized controlled trial
Source: Trials. 2019 Jan 3;20:1. doi: 10.1186/s13063-018-3101-x (PMC6318891; doi:10.1186/s13063-018-3101-x)
Supplement: Supplementary file 1 — SPIRIT checklist. (DOC 117 kb) [file 13063_2018_3101_MOESM1_ESM.doc]

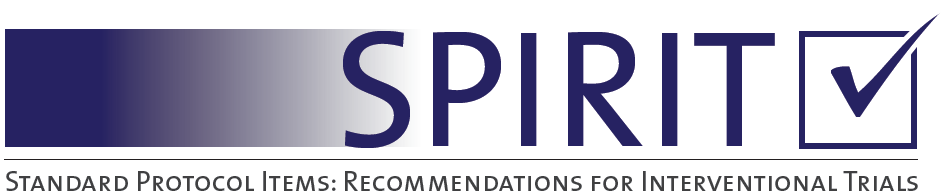


SPIRIT 2013 Checklist: Recommended items to address in a clinical trial protocol and related documents*

| Section/item | ItemNo | Description |
| --- | --- | --- |
| **Administrative information** | | |
| Title | 1 | Page 1, line 1 – line 3 |
| Trial registration | 2a | Page 4, line 63 – line 64 |
|  | 2b | Not applicable |
| Protocol version | 3 | Not discuss |
| Funding | 4 | Page 16, line 341 – line 343 |
| Roles and responsibilities | 5a | Page 1, line 4 – line 17; Page 16, line 344 – line 349 |
|  | 5b | Page 2, line 33 – line 38 |
|  | 5c | Not discuss |
|  | 5d | Not discuss |
| Introduction |  |  |
| Background and rationale | 6a | Page 5, line 80 - Page 6, line 128 |
|  | 6b | Not applicable |
| Objectives | 7 | Page 7, line 133 – line 137 |
| Trial design | 8 | Page 7, line 129 – line 132 |
| Methods: Participants, interventions, and outcomes | | |
| Study setting | 9 | Page 7, line 134 – line 137 |
| Eligibility criteria | 10 | Page 7, line 141 – line 147 |
| Interventions | 11a | Page 8, line 158 – Page 9, line 182 |
|  | 11b | Page 8, line 163 – line 166; line 171 – line 172; Page 9, line 177 – line 179 |
|  | 11c | Not discuss |
|  | 11d | Not discuss. |
| Outcomes | 12 | Page 10, line 206 – line 222, Page 11, line 223 – line 231 |
| Participant timeline | 13 | See Table 1 and Figure 1. |
| Sample size | 14 | Page 11, line 232 – line 243 |
| Recruitment | 15 | Page 7, line 133 – line 140 |
| **Methods: Assignment of interventions (for controlled trials)** | | |
| Allocation: |  |  |
| Sequence generation | 16a | Page 7, line 149 – line 151 |
| Allocation concealment mechanism | 16b | Page 7, line 151 – Page 8, line 156 |
| Implementation | 16c | Page 8, line 155 – line 156 |
| Blinding (masking) | 17a | Not applicable |
|  | 17b | Not applicable |
| **Methods: Data collection, management, and analysis** | | |
| Data collection methods | 18a | Not discuss |
|  | 18b | Not discuss |
| Data management | 19 | Not discuss |
| Statistical methods | 20a | Page 12, line 245 – line 256 |
|  | 20b | Not discuss |
|  | 20c | Not discuss |
| **Methods: Monitoring** | | |
| Data monitoring | 21a | Not discuss |
|  | 21b | Not discuss |
| Harms | 22 | Not discuss |
| Auditing | 23 | Not applicable |
| Ethics and dissemination | | |
| Research ethics approval | 24 | Page 15, line 333 – line 335 |
| Protocol amendments | 25 | Not discuss |
| Consent or assent | 26a | Not applicable |
|  | 26b | Page 15, line 336 – line 337 |
| Confidentiality | 27 | Not discuss |
| Declaration of interests | 28 | Page 16, line 340 |
| Access to data | 29 | Page 15, line 338 – line 339 |
| Ancillary and post-trial care | 30 | Not applicable |
| Dissemination policy | 31a | Not applicable |
|  | 31b | Not applicable |
|  | 31c | Not discuss |
| Appendices |  |  |
| Informed consent materials | 32 | Not discuss |
| Biological specimens | 33 | Not applicable |

*It is strongly recommended that this checklist be read in conjunction with the SPIRIT 2013 Explanation & Elaboration for important clarification on the items. Amendments to the protocol should be tracked and dated. The SPIRIT checklist is copyrighted by the SPIRIT Group under the Creative Commons “[Attribution-NonCommercial-NoDerivs 3.0 Unported](http://www.creativecommons.org/licenses/by-nc-nd/3.0/)” license.
